# Supplementary material for: The Neuropathological Diagnosis of Alzheimer’s Disease—The Challenges of Pathological Mimics and Concomitant Pathology
Source: Brain Sci. 2020 Jul 24;10(8):479. doi: 10.3390/brainsci10080479 (PMC7463915; doi:10.3390/brainsci10080479)
Supplement: Supplementary file 1 [file brainsci-10-00479-s001.pdf]

Supplementary Materials: The Neuropathological Diagnosis of Alzheimer's Disease—The Challenges of Pathological Mimics and Concomitant Pathology

Andrew King , Istvan Bodi and Claire Troakes

Table S1. Table to illustrate the Braak and Braak Staging for Neurofibrillary Tangles (NTs) and Threads in Alzheimer's disease (after Braak and Braak [8]).

Stage	Regions	Distribution of neurofibrillary tangles And threads
I	Entorhinal	Transentorhinal and entorhinal
II		Above and CA1 region of hippocampus
III	Limbic	Above and Subiculum of hippocampus
IV		Above and Amygdala, thalamus and claustrum
V	Isocortical	Above and Associative cortical areas e.g. parastriate cortex
VI		Above and primary visual areas e.g. calcarine cortex

Table S2. Modified Braak/Brain Net Europe neurofibrillary staging system for AD (after Alafuzoff et al. [9]).

**Stage**	**Regions of brain affected**	**affected and density of neuropil threads (Nths)**
**Stage 0**	All sections	No HP-Tau
Stage +	Any section	Very occasional tangles, pretangles and Nths and not in any pattern not fit to any other tauopathy
Stage I	Anterior hippocampus at uncus level	NThs of at least low density in transentorhinal region
Stage II	Anterior hippocampus at uncus level	NThs of at least moderate density outer layers of entorhinal region; at least low density inner layers of entorhinal region
	Posterior hippocampus at level of lateral geniculate body	Nths of at least moderate density outer layers of entorhinal; at least low density inner layers of entorhinal region
Stage III	Anterior hippocampus at uncus level	NThs of at least moderate density in superficial and deep layers of the occipito-temporal gyrus.
	Posterior hippocampus at level of lateral geniculate body	NThs of at least moderate density in outer and inner layers of entorhinal region and continuing to occipito-temporal gyrus.
Stage IV	Severe involvement of middle temporal gyrus	NThs of at least moderate density in superficial and/or deep layers middle temporal gyrus

Stage V		NThs of at least moderate density in superficial and deep layers of peristriate (and parastriate) cortex
Stage VI	Severe involvement of occipital cortex	NThs of at least moderate density in layer V of the striate area.

Table S3. CERAD score for neuritic plaques (after Mirra et al. [10]).

Age related plaque score:.

**Age**	**Frequency of Plaques**			
	**None**	**Sparse**	**Moderate**	**Frequent**
<50	0	C	C	C
50-75	0	B	C	C
>75	0	A	B	C

0-No Histological evidence of AD
A-Histological findings are uncertain evidence of AD
B-Histological findings suggest the diagnosis of AD
C-Histological findings indicate the diagnosis of AD

Minimum six areas of cerebrum sampled : Middle frontal gyrus, superior and middle temporal gyri, anterior cingulate gyrus, inferior parietal lobule, hippocampus and entorhinal cortex, midbrain including nigra.

Neuritic plaque semi-quantitative count (silver stain, or HP-tau) Aβ does not detect neuritic elements.

Table S4. Thal phases for brain parenchymal Aβ deposition (Present or absent) (after Thal *et al.* [11]).

**Phase 1**	**Isocortical e.g. frontal cortex , occipital cortex**
Phase 2	Above and allocortical deposits e.g. entorhinal cortex, hippocampus, amygdala
Phase 3	Above and subcortical nuclei e.g. striatum and thalamus
Phase 4	Above and brainstem structures e.g. substantia nigra
Phase 5	Above and molecular layer of cerebellum or pons
